# Trends in clinical trials for articular cartilage repair by cell therapy

**DOI:** 10.1038/s41536-018-0055-2

**Published:** 2018-10-11

**Authors:** Takaharu Negoro, Yuri Takagaki, Hanayuki Okura, Akifumi Matsuyama

**Affiliations:** 1 Department of Regenerative Medicine Support Promotion Facility, Center for Research Promotion and Support, Fujita Health University, 1-98, Dengakugakubo, Kutsukake-cho, Toyoake, Aichi 470-1192 Japan; 2 Office of Policy and Ethics Research, National Institutes of Biomedical Innovation, Health and Nutrition, 7-6-8, Saito Asagi, Osaka, Ibaraki 567-0085 Japan; 3 Department of Regenerative Medicine, School of Medicine, Fujita Health University, 1-98, Dengakugakubo, Kutsukake-cho, Toyoake, Aichi 470-1192 Japan

## Abstract

Focal and degenerative lesions of articular cartilage greatly reduce the patient’s quality of life. Various therapies including surgical treatment have been developed, but a definitive therapy is not yet known. Several cell therapy products have already been developed and are available in the market. In this study, we examined the clinical research trends related to cell therapy products in the cartilage repair field based on data obtained from the ClinicalTrial.gov website. Although this website does not provide comprehensive results of clinical trials, it offers information on prospective clinical trials, including work in progress, and thus allows for chronological analysis of the data. We selected 203 studies related to the field of cartilage regeneration from ClinicalTrial.gov. The results showed a shift in the clinical translational trend in utilized cells from cartilage- and bone marrow- to adipose tissue-based cells. Whereas the studies that used cartilage as the cell source included many phase III trials, fewer studies using bone marrow and adipose tissue cells progressed to phase III, suggesting that most clinical developments using the latter sources have not been successful so far. One product covered the entire period from the start of phase I to the completion of phase III, with a time to completion of more than 100 months. Translational trends in autologous chondrocyte implantation were also discussed. The use of ClinicalTrials.gov as the sole data source can yield a perspective view of the global clinical translational trends, which has been difficult to observe up to this point.

## Cartilage repair: temporal shift in source of cell therapies

Bone marrow and fat tissue are increasingly tested, although the most clinically advanced cell therapies are still derived from cartilage. Takaharu Negoro from Fujita Health University in Toyoake, Japan, and colleagues identified 203 prospective trials involving cell therapy products for articular cartilage repair. They examined the sources of the cells using a unique chart analysis and observed a shift over time from studies testing products derived from cartilage and bone marrow to, most recently, those from fat. As the researchers document, however, most cell therapies that are approved or in late-stage testing come from cartilage cells, usually a patient’s own. Few of the newer candidates from bone marrow or fat tissue have successfully progressed from phase II proof-of-concept studies to phase III efficacy trials.

## Introduction

ClinicalTrials.gov^1^ is the clinical trial registration database of the United States, which provides information on the implementation status of more than 260 000 clinical trials from over 200 countries and is the world’s largest clinical trial registration site. Although ClinicalTrials.gov does not provide comprehensive results of clinical trials, it is a database of the plans for individual trials and provides information about target diseases, sponsors/principal investigators, planned schedule, and protocols of the clinical trials and enrollment of the subjects. Furthermore, since the database provides comprehensive information on the details of the content of the planned clinical trial, one can perform various targeted analyses by extracting and tagging attribute data from each clinical study plan.

Focal and degenerative lesions of articular cartilage greatly reduce a patient’s quality of life. Various therapies including surgical treatment have been developed, but a definitive therapy is not yet known. Investigators have attempted to repair cartilage defects by using cell therapy since the end of the last century. In fact, there are already several early cell therapy commercial products on the market in the United States, Europe, Korea, and Japan.^2–7^ Several excellent reviews have been published of products developed in this field so far.^8–15^ However, to the best of our knowledge, there are no scientific reports that have comprehensively analyzed and examined the clinical research trends on cell therapy for articular cartilage regeneration based on the ClinicalTrials.gov data registry. In this article, focusing on cell therapy products for cartilage repair, which require manufacturing and marketing approval by national authorities, based on the data obtained from ClinicalTrials.gov, we aimed to grasp a big picture of the global translational trend, which has thus far been difficult to decipher.

## Results

**Fig. 1:**
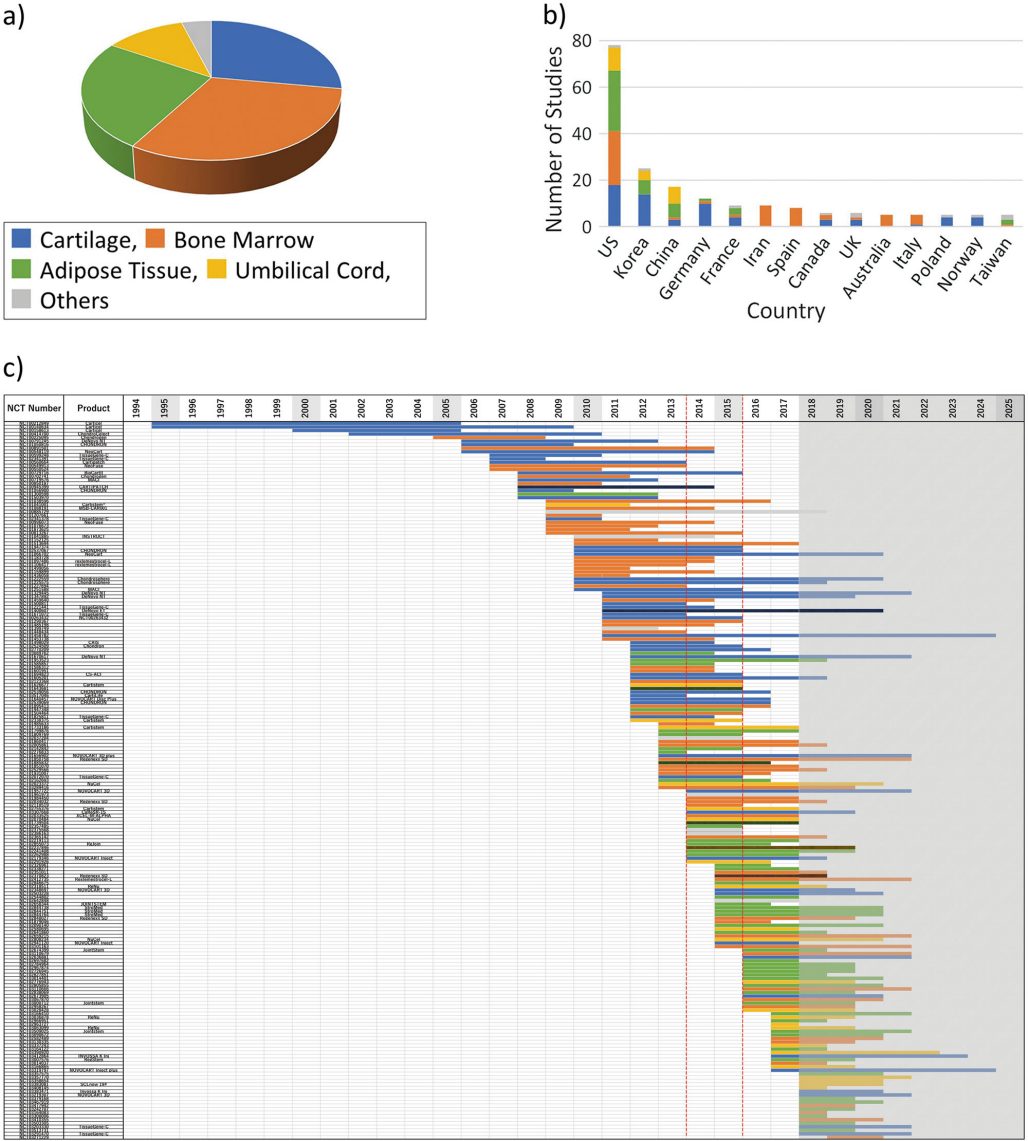
Analysis of projects in ClinicalTrials.gov according to the cell source organ used for cell therapy and cartilage repair. “Others” include studies that are using multiple cell sources for combination or comparison. **a** Percentage of each cell source relative to the total number of studies. **b** Comparison of number of clinical trials on cartilage repair according to countries of origin. Each color-coded part of the bar depicts the corresponding cell-source organ by country. The top 12 countries are shown in this graph. **c** Each study was color-coded by the corresponding cell source organ and displayed from the start year to the (planned) completion year, sorted by start year in chronological order. Shaded column: current year (2018) to 2025. Since 2018, a trial bar displays if the trial is registered. Please note that we could not show if the trial continued or was halted prematurely. Red-dashed column: 2014–15. Vertical-striped bar indicates “suspended,” “terminated,” or “withdrawn” study

We surveyed the website ClinicalTrials.gov and selected 203 studies on regenerative cartilage repair. Using the retrieved data, we then analyzed the translational trends described in these studies. First, we classified the entire list of studies by the cell source organ used. The results are shown in Fig. 1a. The major organs used were as follows: bone marrow (31%), cartilage (28%), adipose tissue (25%), and umbilical cord (12%).

Figure 1b shows the analyzed results by country. The United States, which manages ClinicalTrials.gov, had 79 studies, ranking at the top. In second place was Korea, followed by China, Germany, France, Iran, and Spain. In the same graph, each color-coded bar depicts the corresponding source of cells by country. The US studies used mainly cartilage, bone marrow, and adipose tissues at a rate of approximately 1:1:1 as the cell source. This rate varied among the listed countries. For example, Korea and Germany used mainly cartilage, while Iran, Spain, and Australia used bone marrow only.

To analyze the clinical research trends described in these projects chronologically, we arranged all studies by order of the corresponding start year, plotted them from the start year to the completion year, and color-coded them according to the organs of cell origin (Fig. 1c). This analysis showed that cartilage was used as a source of cells from the beginning of 1995 to the present, but the rate of use decreased from an average of 45% (2006–2012) to 10–15% (after 2013). On the other hand, bone marrow has been used since 2009, followed by adipose tissue since 2012. The total number of studies using umbilical cord is small, but this source of cells has been implemented since 2009. The above trends have continued to date. Interestingly, a clinical trend of cell-derived tissues shifting from cartilage and bone marrow to adipose tissue has been observed since 2014–2015. Collectively, these findings indicate that the clinical application of cartilage repair began in the mid-1990s with the use of cartilage tissue as the cell source, and bone marrow has also been studied since the mid-2000s, but in the mid-2010s, both were replaced mainly with adipose tissue.

**Fig. 2:**
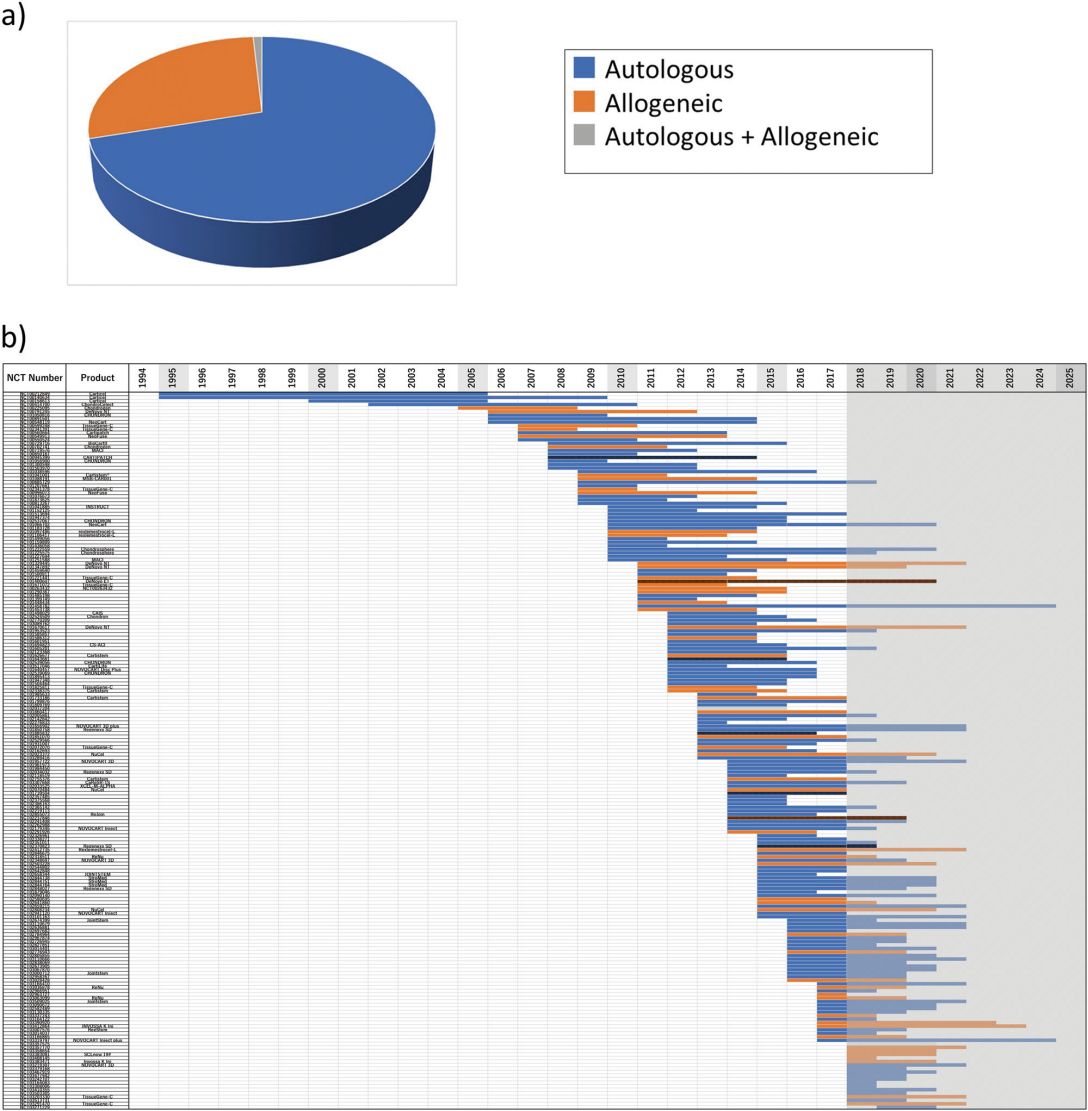
Analysis of origin of cells (autologous or allogeneic) used for cell therapy and cartilage repair in clinical trials registered in ClinicalTrials.gov. **a** Percentage of each origin of cells relative to the total number of studies. **b** Each study is color-coded by corresponding origin of cells and displayed from the start year to the (planned) completion year, sorted by start year in chronological order. Shaded column: current year (2018) to 2025. Since 2018, a trial bar displays if the trial is registered. Please note that we could not show if the trial continued or was halted prematurely. Vertical-striped bar indicates “suspended,” “terminated,” or “withdrawn” study

In the next step, the origin (autologous or allogeneic) of each cell source was analyzed (Fig. 2a). Studies that used cells of allogeneic origin comprised approximately one-third of the entire database. Overall, no specific chronological trend was observed for either origin (Fig. 2b).

Table 1 provides a detailed list of the cell therapy products designed for cartilage repair that are approved by the regulatory authorities of various countries and are currently available in the market. Briefly, Carticel®^2^ was a first-generation autologous chondrocyte implantation (ACI) product, which required open arthrotomy implantation of in vitro-cultured autologous chondrocytes beneath an autologous periosteal cover. ChondroCelect®^8^ was also a first-generation ACI product utilizing a proprietary genetic marker profile score that optimizes the likelihood of a hyaline phenotype and its associated biological, cartilage-forming capability. MACI®^3,4,8,9^ is a type I/III collagen membrane seeded with expanded autologous chondrocytes. Spherox (chondrosphere®^16^) consists of small spheroids of neocartilage composed of expanded autologous chondrocytes and their associated matrix. Chondron™^5^ is an autologous chondrocyte-pre-seeded fibrin three-dimensional matrix gel. CARTISTEM®^6^^17^ is a composite of allogeneic umbilical blood mesenchymal stem cells (MSCs) and hyaluronic acid hydrogel. Invossa™^18^ (TissueGene-C) is a gene therapy implant that includes modified transforming growth factor-β (TGF-β)-expressing allogeneic chondrocytes.^19^ JACC is cultured autologous chondrocytes embedded in atelocollagen gel.^7^ Although MACI® was initially approved by European Medicines Agency (EMA), it was suspended in the European Union (EU) in 2014 because of a manufacturing site closure in Europe.^20^ Furthermore, ChondroCelect® was withdrawn from the market in 2016 because of a reimbursement problem in the EU.^21^ Carticel® was phased out because of the new approval of MACI® in the United States.^22^ On the other hand, two new products, Spherox^16^ and Invossa™^18^ were recently approved in the EU and Korea, respectively. Both are derived from cartilage as the cell sources. As shown in Table 1, most of the globally marketed products for cartilage repair (with the exception of CARTISTEM®^6^) are derived from cartilage. Unfortunately, the available data on the registered studies in ClinicalTrials.gov do not allow for direct estimation of the market share of each type of product.

**Table 1:** List of cell therapy products used in cartilage repair area available on the world market

Product name	Company	Country	Approval	Indication	Remarks
Carticel®	Vericel	US	1997 FDA	Cartilage defects of the femoral condyle (medial, lateral, or trochlea)	First ACI/2017 phased out
MACI®	Vericel	US	2016 FDA	Full-thickness cartilage defects of the knee	Second ACI
ChondroCelect®	TiGenix	EU	2009 EMA	Cartilage defects of the femoral condyle of the knee	First ACI/2016 withdrawn
MACI®	Vericel	EU	2013 EMA	Full-thickness cartilage defects of the knee	Second ACI/2014 suspended
Spherox (chondrosphere®)	co.don	EU	Jul 2017 EMA	Articular cartilage defects of the femoral condyle and knee patella	Third ACI
Chondron™	Sewon Cellontech	Korea	2001/2008 MFDS	Articular cartilage defect (knee/ankle)	Second ACI
CARTISTEM®	Medipost	Korea	2012 MFDS	Osteoarthritis due to degeneration or repetitive trauma	Allogeneic umbilical cord blood-derived MSC drug
Invossa™ (TissueGene-C)	Kolon Life Sciences	Korea	Jul 2017 MFDS	Moderate knee osteoarthritis with persistent symptoms despite ≥3 months of conservative treatment	TGF-β-expressing allogeneic chondrocytes
JACC	Japan Tissue Engineering	Japan	2012 MHLW	Traumatic arthritis, osteochondritis dissecans of the knee	Second ACI

The list includes marketed products announced by each national regulatory authority as of 1 August 2017. For the United States, the FDA-approved products are listed, but not 361 HCT/P products. For the European Medicines Agency (EMA), the EMA-approved products are listed, but not the hospital exemption products. For Korea, MFDS-approved products are listed. In Japan, the MHLW-approved products are listed

**Fig. 3:**
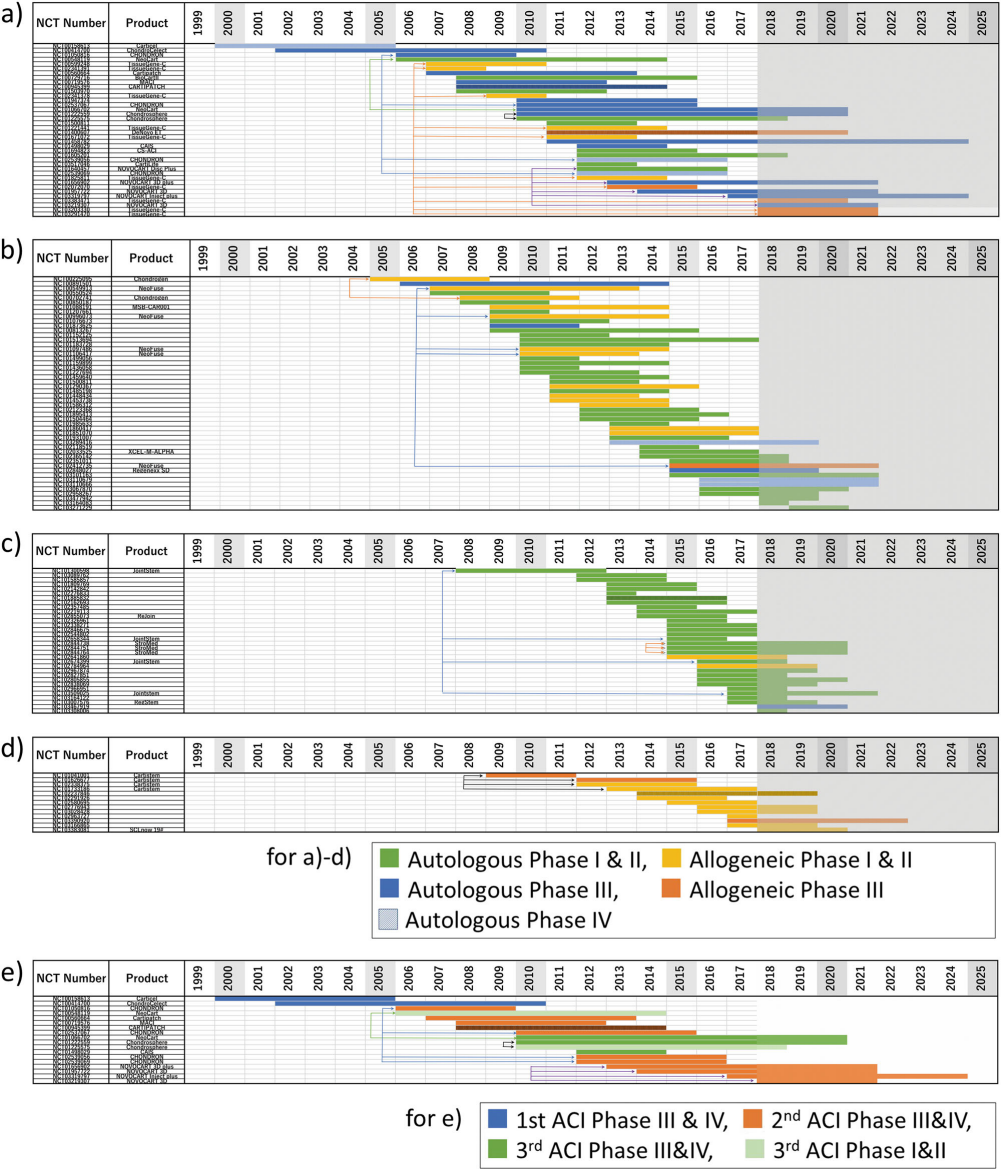
Chronological display (sorted by start year) of cartilage repair trials in which **a** cartilage, **b** bone marrow, **c** adipose tissue, or **d** umbilical cord^7^ was used as the cell source. Each study was plotted from start year to completion year as a color-coded bar showing the origin of the cell source (autologous or allogeneic) and the corresponding clinical stage, as shown in examples in the frame below **d**. For convenience, trackable products with multiple trials were linked with colored lines and arrows as follows; **a** blue: Chondron, green: NeoCart, yellow: TissueGene-C, black: Chondrosphere, and purple: Novocart; **b** blue: NeoFuse and yellow: Chondrogen; **c** blue: JointStem and yellow: StroMed; **d** black: CARTISTEM. **e** Chronological display (sorted by start year) of the clinical trials of each generation of ACI products. Each study was plotted from the start to the completion year as a color-coded bar, which indicates the generation of ACI and corresponding clinical phases, as shown in examples in the frame below **e**. We could not find any phase I and II studies corresponding to the first ACI in ClinicalTrials.gov. Shaded column: current year (2018) to 2025. Since 2018, a trial bar displays if the trial is registered. Please note that we could not show if the trial continued or was halted prematurely. Vertical-striped bar indicates “suspended,” “terminated,” or “withdrawn” study

The origin of the cell source (autologous or allogeneic) and the clinical stage were analyzed chronologically to determine progress in testing new products derived from each of the four main cell sources (bone marrow, cartilage, adipose tissue, and umbilical cord). Although not shown in Fig. 3a because of the lack of description of the phase of trial, the earliest cartilage cell therapy trials in ClinicalTrials.gov started in 1995 (Fig. 1c). As shown, cartilage has been examined as a material for cell therapy for cartilage repair for a long time. The start of phase III clinical trials on a newly developed product reflects positive results in phase II studies with regard to its effectiveness. Interestingly, a large proportion (15%) of phase III clinical trials for cartilage repair registered in the ClinicalTrials.gov database was included in this group, most of which used autologous cells (12/15). Therefore, our findings indicate that several earlier clinical studies using autologous cartilage as the cell source have shown encouraging results to warrant phase III clinical trials for cartilage repair. On the other hand, allogeneic cartilage cells were used in 11 studies. Only 4 of these were registered as phase III, but all of them were studies on TissueGene-C (NCT02072070, NCT03203330, NCT03291470, and NCT03383471). TissueGene-C was approved by the Korean authorities in July 2017 and marketed as Invossa™ (Table 1).

Six of the phase III clinical trials using autologous cartilage cells are currently being conducted in 2018. They are examining three products (NeoCart^8^ (NCT01066702), chondrosphere® (NCT01222559), and Novocart 3D^8^ (including 3D plus, and Inject plus) (NCT01656902, NCT01957722, NCT03219307, NCT03319797, and NCT03383471)), all of which are classified as ACI. Among them, Chondrosphere® and Novocart 3D are cell therapies that have already been used clinically under the hospital exemption (HE) scheme in Germany. The HE is a European-specific scheme that grants approval for use of medical products on an experimental basis in specific hospitals, even though the effectiveness of such products remains to be confirmed.^23^ Chondrosphere® was also approved by EMA in July 2017 and marketed as Spherox (Table 1).

Since Wakitani et al.^24^ reported the first case of treatment of cartilage defects with autologous MSCs in 2004, bone marrow has often been used as a source of MSCs. Figure 3b shows the results of studies to repair cartilage defect using bone marrow as the cell source. The earliest study was conducted in 2006. Research employing bone marrow as the source for chondrocytes became active around 2009. Two phase II/III trials (NCT00891501 and NCT01873625) were conducted in the early years (2006 and 2009, respectively), although no information is available regarding their approval. Interestingly, no phase III trials were conducted for several years after the above two studies. One phase III trial using an autologous cell source in 2015 was found, but this study (NCT02848027) was not relevant to our analysis because it was for a 361 HCT/P product, which does not require the approval of the US Food and Drug Administration (FDA).^25^

Although many studies using allogeneic cell sources were also examined in the initial stages, no phase III studies were registered in the database until 2014. While degenerative disc disease (DDD) is not an articular cartilage disease, one phase III clinical trial using rexlemestrocel-L (NeoFuse™)^26^ for DDD in the United States and Australia (NCT02412735) was registered in 2015. In summary, there are no approved cartilage repair products based on clinical trials that used bone marrow-derived cells registered in ClinicalTrials.gov, and the rate of progression to phase III is low (3.2%). Among these studies, there is only one allogeneic product for DDD that is currently under development in a phase III clinical trial.

Adipose tissue has also been used as a source for MSCs. Figure 3c shows the analysis of the projects that used adipose tissue as the cell source. The earliest study was conducted in 2008, but such studies became more common after 2012. There is only one phase III trial among these studies (NCT03467919). Although this study used MSCs extracted from adipose tissue, any earlier corresponding trials were not found in ClinicalTrials.gov. On the other hand, the use of allogeneic cell sources was low (3.9%). Aggressive use of adipose tissue as the cell source for cartilage repair began around 2012 and has been actively studied, but other phase III trials were not found.

The results of the analysis of studies using cells originating from the umbilical cord are shown in Fig. 3d. The data shown in this figure also include studies using cells from Wharton’s jelly, placenta, and amniotic membrane/fluid. All registered studies were conducted after 2008 and included two phase III trials in the early years for CARTISTEM® (NCT01041001 and NCT01626677). A phase II/III trial using amniotic fluid started last year, although we could not find any earlier corresponding trials in ClinicalTrials.gov. All other studies using cell sources classified in this category remain in phase II or earlier phases to date.

We also focused on studies on ACIs that were translationally successful among all clinical trials. Figure 3e includes information on ACI studies that were analyzed for clinical development trends in chronological order. The ACI studies were classified into three generations based on the method described by Harris et al.^8^ Studies on the first-generation ACI were completed by 2010, while the second-generation ACI has been actively studied since 2006 to the present. Furthermore, the third generation was also studied at almost the same time as the second generation. Interestingly, the proportion of phase III trials relative to all trials for each generation was high, suggesting successful development of the technology. Thus, our analysis suggests that the major current trend in clinical development is the second- and third-generation ACI, while the first-generation ACI is superseded technology.

Next, we analyzed the time required for clinical development in this field. For this purpose, we analyzed all products (including candidates) that were used in phase I to phase III and that could be traced by product name or development code. Specifically, we analyzed the time required for a series of studies from phase I to phase III trials. Only two products/three research projects that covered phases I–III were identified in the ClinicalTrials.gov database (Table 2). Among the three projects that were entirely trackable (from the start of phase I to the completion of phase III) in ClinicalTrials.gov, only one project completed in practice was for TissueGene-C in Korea, and the time required to complete this entire project was 103 months. Since the remaining two projects were incomplete at this time (June 2018), it is necessary to be aware that these are projected periods. With regard to the clinical trial on the use of NeoFuse™ for DDD, the estimated time for the completion of phase I–phase III is 150 months.

**Table 2:** List of clinical research series of product candidates that included phases I–III

Product	Country	Phase I start	Phase I complete	Phase II start	Phase II complete	Phase III start	Phase III complete	Approval	Phase I start–phase III complete (months)
TissueGene-C (Invossa™)	Korea	Feb 2007	Sep 2008	Aug 2009	Jun 2014	Nov 2013	Aug 2015	Jul 2017	103
		NCT02341391		NCT02341378 NCT01671072 NCT01825811		NCT02072070			
TissueGene-C	US	Jan 2007	May 2010	May 2011	Oct 2014	Apr 2018	(Jun 2021)	–	(176)
		NCT00599248		NCT01221441		NCT03203330 NCT03291470			
rexlemestrocel-L (NeoFuse™)	US/Australia	Oct 2007	–	Jun 2010	Jul 2015	Mar 2015	(Feb 2020)	–	(150)
		NCT00549913		NCT01097486 NCT01106417 NCT01290367		NCT02412735			

Alternatively, to examine whether there is any tendency in the period required for each trial depending on the combination of cell source and origin, we extracted all the completed trials in practice and classified them according to their cell source and origin, and calculated the actual period taken to complete each study. Studies using autologous cartilage cells reached completion at a median of 74 months, while those using allogeneic cartilage cells were completed within a median of about 26 months, and the difference between these two types was significant. On the other hand, the use of allogeneic bone marrow cells and adipose tissue cells was associated with slightly longer times (median 46 and 36 months, respectively) than those using corresponding autologous cells (median 34 and 32 months), although the difference in these times was not significant. Interestingly, compared to the use of autologous chondrocytes, both the use of autologous bone marrow and autologous adipose tissue was significantly shorter, with a median of 34 and 32 months, respectively.

**Fig. 4:**
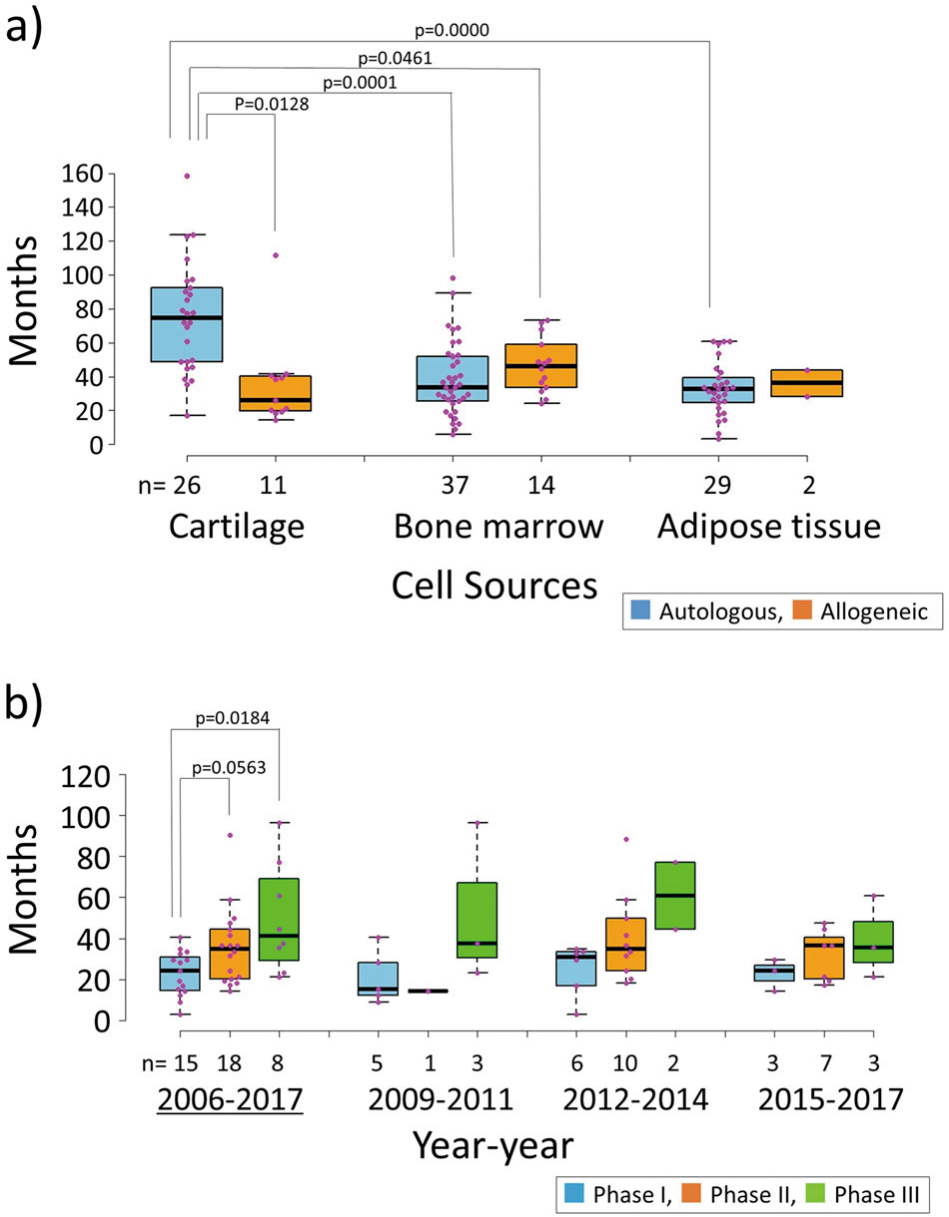
**a** Box-plots show the comparison of periods required for studies using cell sources derived from autologous or allogeneic origin. Blue boxes with whiskers: autologous cells; orange boxes with whiskers: allogeneic cells. Pink-colored dots: period required for individual trials. As Shapiro-Wilk tests revealed that three of six specimens were not normally distributed, Steel-Dwass’ tests were conducted to test the difference between the six specimens. **b** Box-plots show the transition of time required for completion of individual studies completed until 2017 by each phase. The data in the entire period from 2006 to 2017 (underlined) are shown on the left side of this figure, and the transition of the required time by each 3-year period is indicated in the rest. Blue boxes with whiskers: phase I; orange boxes with whiskers: phase II; green boxes with whiskers: phase III. Pink-colored dots: periods required for individual trials. As Shapiro-Wilk tests revealed that one of three specimens was not normally distributed, Steel-Dwass’ tests were conducted to test the difference between the three specimens

Finally, to examine the time required for each phase in more detail, the completed studies registered as phase I, phase II, or phase III were extracted, and we analyzed them by the time required for individual studies using the completion year, instead of the start year. Figure 4b shows the median time required for completion of individual studies completed by 2017 by each phase as boxplots. In the entire period, the phase I trials were completed in a median of 24 months, the phase II trials in a median of 36 months, and the phase III trials in a median of 41 months. Moreover, significant differences were observed in the required times between the phase I and phase III studies, and a difference in those between phase I and phase II studies was nearly significant (*p* = 0.0563). The simple summation of the median time required to complete each phase was 102 months. As few corresponding studies were completed by 2008, the period from 2009 to 2017 was divided into three sections, which are shown in the figure, to reveal the transition every 3 years. Although statistical analysis was impossible because of the small number of samples, it was found that the median duration of phase I studies was 15–31 months, 14–35 months for phase II studies, and 35–61 months for phase III studies.

## Discussion

There are already two interesting studies that provided comprehensive analysis relevant to regenerative medicine based on data from ClinicalTrials.gov. Monserrat et al.^27^ analyzed the entire disease field with a special focus on stem cells and provided comprehensive information on the global trend of translational research, but unfortunately, their research did not include any detailed information on the individual fields. On the other hand, Fung et al.^28^ provided a comprehensive assessment of the extent to which the publication of results of clinical trials of innovative cell-based interventions reflects the best practice guidelines of the International Society for Stem Cell Research and discussed various ethical considerations. Apart from these studies, to date, there are no clear reports on comprehensive clinical development trends in specific fields of regenerative medicine using the clinical trial registry. In this study, we focused on cell therapy applied so far for cartilage repair and used data available on the ClinicalTrials.gov registry as the primary source to conduct comprehensive and chronological research, classification, and analysis of clinical trials registered in this field, including world research trends on cell therapy.

The reason why ClinicalTrial.gov is not used for research as a sole information source is because this database does not provide comprehensive results of clinical trials, and it is impossible to analyze the results of the trials by themselves. That is, in order to review and analyze clinical development in a certain field, it is necessary to obtain the result data from another information source. The above two papers could avoid this drawback. This study aimed to comprehensively analyze cell therapy in the cartilage repair field, but unlike ordinary reviews, we decided to use it as a database of the trial plan, ignoring results in ClinicalTrials.gov. In other words, we utilized this planning database of clinical trials to capture the big trends in translational studies in this field by comprehensive chronological analysis. By performing attribute analysis using chronological display, we were able to obtain a perspective view (Figs. 1c, 2b, and 3a–e). The increase in the number of registered studies since the mid-2000s is thought to be due to the International Committee of Medical Journal Editors (ICMJE) and/or the FDA Amendments Act of 2007, which promoted registration. The data shown in Fig. 1c indicate that cartilage repair therapy started originally using autologous cartilage tissue as the cell source. This was followed by the use of cells from the bone marrow and adipose tissue, as well as other tissues. Based on this analysis, the results showed a shift in the selected tissue from cartilage and bone marrow to adipose tissue in 2014–2015.

In clinical trials registered in ClinicalTrials.gov, bone marrow was the most popular cell source in this field (Fig. 1a, c), but there are no products on the market at present that utilize these cells. Our investigation revealed that most of the commercial products were derived from cartilage (Table 1). In the field of cartilage repair, any cell therapy products prepared from cells other than cartilage and umbilical cord have not yet been approved by any national authorities. Comparison of the use of each cell source at the clinical translational stage showed a high proportion of phase III using cartilage as the cell source (Fig. 3a), compared with a low rate of those of bone marrow-derived cells (Fig. 3b). On the other hand, the registry contained only one phase III study using adipose tissue (Fig. 3c). Worth nothing, the start year of translational studies using cartilage as a cell source was almost more than 10 years earlier than the others. In 2004, Wakitani’s article triggered further research on bone marrow as a cell source. Also, adipose tissue has been used as a source of MSCs since 2008. In other words, since the start of the translational study for both bone marrow and adipose tissue was substantially slower than that of cartilage (Fig. 3a–c), the time might have been insufficient to reach phase III. Even in the trials using cartilage, phase III trials using autologous cells were quite popular, while those using allogeneic cells were not. One reason is due to the difference in start year of the translational research, as described above. To use autologous cartilage, it is necessary to collect cartilage for expanding culture beforehand, but the first operation is not necessary for using allogeneic cells. Despite such benefits, highly invasive surgical techniques such as conventional microfractures have been established as a standard therapy using autologous cartilage so far, and on the extended line of such procedures, cell therapy requiring autologous cartilage collection might have been relatively easily to accept. Using autologous cartilage, two new products (Neocart and Novocart) were examined in phase III. On the other hand, despite the benefits described above, allogeneic products need to be expanded in culture even more than autologous cells and require more strict quality control. It is important that TissueGene-C, which is the only approved product using allogeneic cartilage, is a transgene product expressing TGF-β.

According to Dewan et al.,^14^ cartilaginous tissues regenerated from bone marrow MSCs are fibrocartilaginous and inferior to the original cartilage. On the other hand, a recent review reported that the quality of the regenerated tissue varies according to the clinical trial.^29^ With regard to Provenge® (sipuleucel-T), which is a product for immunotherapy, Galipeau^30^ reported a lack of activity of bone marrow MSCs in a large clinical trial because of the heterogeneity of donors and senescence due to long cultivation. Martin et al.^31^ pointed out that the vision of producing MSCs based on a unique standard is not yet scientifically achievable. Thus, there seems to be certain difficulties in large-scale clinical trials using bone marrow MSCs. In the case using autologous cells, it is concerning that there might be a large influence of inter-individual differences between donors as cell sources. On the other hand, allogeneic products are advantageous because one can choose good cell source(s) among donor candidates. Especially, umbilical cord blood-derived products are more advantageous because of younger cell source(s). Although the first study using umbilical cord cells was found in ClinicalTrials.gov on 2009 (Fig. 3d), this was a phase III study in Korea of CARTISTEM®, which has already been marketed in Korea since 2012. We thought that these factors described above, in addition to the positive attitude toward cell therapy from the Korean government at an early stage, may have contributed the translational success of CARTISTEM® in Korea.

In the present study, statistical analysis of the time required for the development of products (from the start of phase I to the completion of phase III) based on the data from the ClinicalTrials.gov registry (Table 2) was not possible because of the small number of samples. However, the obtained data showed that TissueGene-C development was completed in 103 months (about 8.5 years). Regarding the time period required to complete the relevant clinical trial, Kaitin and DiMasi^32^ reported that the time from Investigational New Drug filing to New Drug Application/Biologic License Application submission for new drugs was 6.5 years on average. In comparison, our data, though limited, suggest that a longer time may be required to develop cell therapy products for cartilage repair. In this regard, approval of TissueGene-C, which can be potentially used for gene therapy, may require a longer time to pass various regulatory bodies. These kinds of hurdles are unavoidable, especially for the leading runners in this new field.

Analysis of the time required for individual studies based on cell source and cellular origin (autologous or allogeneic) revealed that the studies using autologous cartilage tissue took more than a twofold-longer period than the studies using the other cell sources and allogeneic cells, as shown in Fig. 4a. Using autologous cartilage, the proportion of phase III trials is higher than other cell sources (compare Fig. 3a with Fig. 3b–d) and the period required for phase III trials is longer than that of phases I and II (from Fig. 4b). Thus, we thought that these were the reasons why a relatively longer period was needed for completing trials using autologous cartilage as a cell source.

Figure 4b shows the transition of the time required to complete an individual study, summarized in the year each study was completed, not the year in which each the study was initiated. Since only the completed studies were analyzed, the number of studies was not sufficient for statistical analysis when they were divided by every 3 years. However, in the whole period (2006–2017), the number was sufficient to try statistical analysis, and the trend of time required for each individual phase (phase I < phase II < phase III) was considered reasonable. The results for the entire period showed that simple summation of the median time of each phase resulted in 102 months to complete all phases. This is equivalent to 8.5 years and is in accordance with the period observed for the clinical development of TissueGene-C in Korea (103 months) shown in Table 2. In the results for 2015–2017, a 6-month decrease was observed. This is a favorable trend for clinical development, although it is necessary to keep in mind that the completion of each phase still required more than 90 months (7.5 years).

The data shown in Fig. 3e suggest that the major trend in the clinical development of products at present is the use of the second- and third-generation ACI after the replacement of the first-generation ACI. Harris et al.^8^ reported that complications, reoperations, and failures were common after first-generation ACI. In this context, TiGenix withdrew ChondroCelect® from the European market in November 2016.^21^ To date, many developers have focused on obtaining regulatory approval. For this reason, it was surprising that TiGenix abandoned the first approval obtained from the EMA. In the case of ChondroCelect®, since it received the first approval from the EMA, TiGenix was able to market the product for the next 7 years. However, as mentioned earlier, the first-generation ACI has already been replaced by newer technology. Thus, because ChondroCelect® was approved as the earliest ATMP in Europe and was on the market for 7 years thereafter, it is thought that ChondroCelect® was successful as a leading cell-therapy product and might have ended its historical role in cartilage regeneration. In addition, since Vericel received FDA approval of MACI® in 2016, another first ACI product, Carticel®, was phased out in 2017.^22^

The problem here is determining whether or not the marketing period of 7 years (as in the case of ChondroCelect®) was long enough in terms of the total investment. As mentioned above, the average time required for the completion of the entire project (i.e., from the start of phase I to the end of phase III) was 103 months for TissueGene-C and 150 months for NeoFuse™. For conventional drug research and development, the time period from basic research to preclinical studies, and the time from the completion of phase III to approval must be added. Because no sales can be made during the research period, it is necessary to estimate prospective profit after the product is given the approval for marketing, taking into consideration the cost of running the entire project.

In essence, it is crucial to shorten the time required for clinical development to accelerate the development of regenerative medicine products. In this sense, conditional approval, as typified by the PMD Act in Japan,^33^ can reduce at least some of the time burden in the clinical development of regenerative medicine products. Based on the PMD Act, if the efficacy is presumed and the safety is confirmed, conditional, and time-limited marketing approval (5 years, conducting post-marketing efficacy studies) can be obtained.^34^

In conclusion, our analysis of the clinical trials registered at the ClinicalTrials.gov website showed that the clinical trend in the use of cells in research has shifted from cartilage- and bone marrow- to adipose tissue-based cells. Whereas studies using cartilage as the cell source included many phase III trials, fewer studies using bone marrow and adipose tissue cells progressed to phase III, suggesting that most clinical developments using the latter sources have not been successful so far. Furthermore, all products approved by the authorities have been those that used cartilage as the cell source, except for one product that used cells from umbilical cord blood.

The time required for the development of such products (from start of phase I to completion of phase III) was more than 100 months. No doubt this period of time is long and may deter future investment in the manufacturing of such products. We believe that the conditional approval system legislated through the Japan PMD Act can help reduce, at least in part, the development time burden and encourage investment in future research and development. The attribute analysis based on the chronological display used in this study seems useful in providing supportive perspective viewpoints.

## Methods

We searched the entire database at ClinicalTrials.gov on 8 May 2018 using the following search terms: “stem cell” OR “regenerative” OR “cell therapy” OR “implant” OR “transplant osteoarthritis” OR “cartilage injury” OR “cartilage repair” OR “Osteochondral Defects” OR “Articular Cartilage” OR “Traumatic Arthritis” OR “cartilage disease” OR “cartilage defect” OR “chondrocyte”. Among the identified 749 studies, we excluded studies using only surgical procedures, low-molecular-weight drugs, protein drugs, or scaffolds by carefully reading the descriptions of the individual studies, and selected 181 studies corresponding to cell therapy, which administered cells to humans to examine their safety and efficacy. Furthermore, the relevant studies were re-surveyed using the product name, development code, and/or sponsor’s name described in the 181 studies as search terms and were selected manually. Twenty-two studies were found to be incorporated into the above previous studies. Accordingly, we selected 203 studies and used their content for analysis.

We recorded the cell source organ, product name (if any), and country where the clinical study was performed. We did not include protocols on the registry that contained minimal information or incomplete data. The cell sources used were classified as cartilage, bone marrow, adipose tissue, umbilical cord, and others. Unspecified mesenchymal progenitor cells and MSCs were included in the “bone marrow” set according to the general usage. “Adipose tissue” included all materials described as adipose and fat. The “umbilical cord” set contained stem cells derived from umbilical cord blood, Wharton’s jelly, placenta, and amniotic membrane/fluid. All cell sources classified as umbilical cord were regarded as “allogeneic”.

To estimate the time of clinical development, we chose projects that were traceable with product name or development code, and calculated the time required for clinical trials using these individual products.

Normality tests were conducted using Shapiro-Wilk test in IBM SPSS Statistics v23. Steel-Dwass’ tests, a nonparametric multiple comparison method, were performed using pSDCFlig in NSM3 package of R v3.4.4. A *p*-value (asymptotic) <0.05 was considered to be statistically significant.

## Data Availability

The data that support the findings of this study are available from 10.6084/m9.figshare.6964715.
